# Autophagy as a new player in the regulation of clock neurons physiology of *Drosophila melanogaster*

**DOI:** 10.1038/s41598-024-56649-3

**Published:** 2024-03-13

**Authors:** Kornel Szypulski, Aleksandra Tyszka, Elzbieta Pyza, Milena Damulewicz

**Affiliations:** Department of Cell Biology and Imaging, Institute of Zoology and Biomedical Research, Faculty of Biology, Jagiellonian University, Krakow, Poland

**Keywords:** Circadian clock, Sleep, Neuroplasticity, The fruit fly, Autophagy, Circadian rhythms, Cellular neuroscience, Circadian rhythms and sleep, Synaptic plasticity

## Abstract

Axonal terminals of the small ventral lateral neurons (sLNvs), the circadian clock neurons of *Drosophila*, show daily changes in their arborization complexity, with many branches in the morning and their shrinkage during the night. This complex phenomenon is precisely regulated by several mechanisms. In the present study we describe that one of them is autophagy, a self-degradative process, also involved in changes of cell membrane size and shape. Our results showed that autophagosome formation and processing in PDF-expressing neurons (both sLNv and lLNv) are rhythmic and they have different patterns in the cell bodies and terminals. These rhythmic changes in the autophagy activity seem to be important for neuronal plasticity. We found that autophagosome cargos are different during the day and night, and more proteins involved in membrane remodeling are present in autophagosomes in the morning. In addition, we described for the first time that Atg8-positive vesicles are also present outside the sLNv terminals, which suggests that secretory autophagy might be involved in regulating the clock signaling network. Our data indicate that rhythmic autophagy in clock neurons affect the pacemaker function, through remodeling of terminal membrane and secretion of specific proteins from sLNvs.

## Introduction

**Figure 1:**
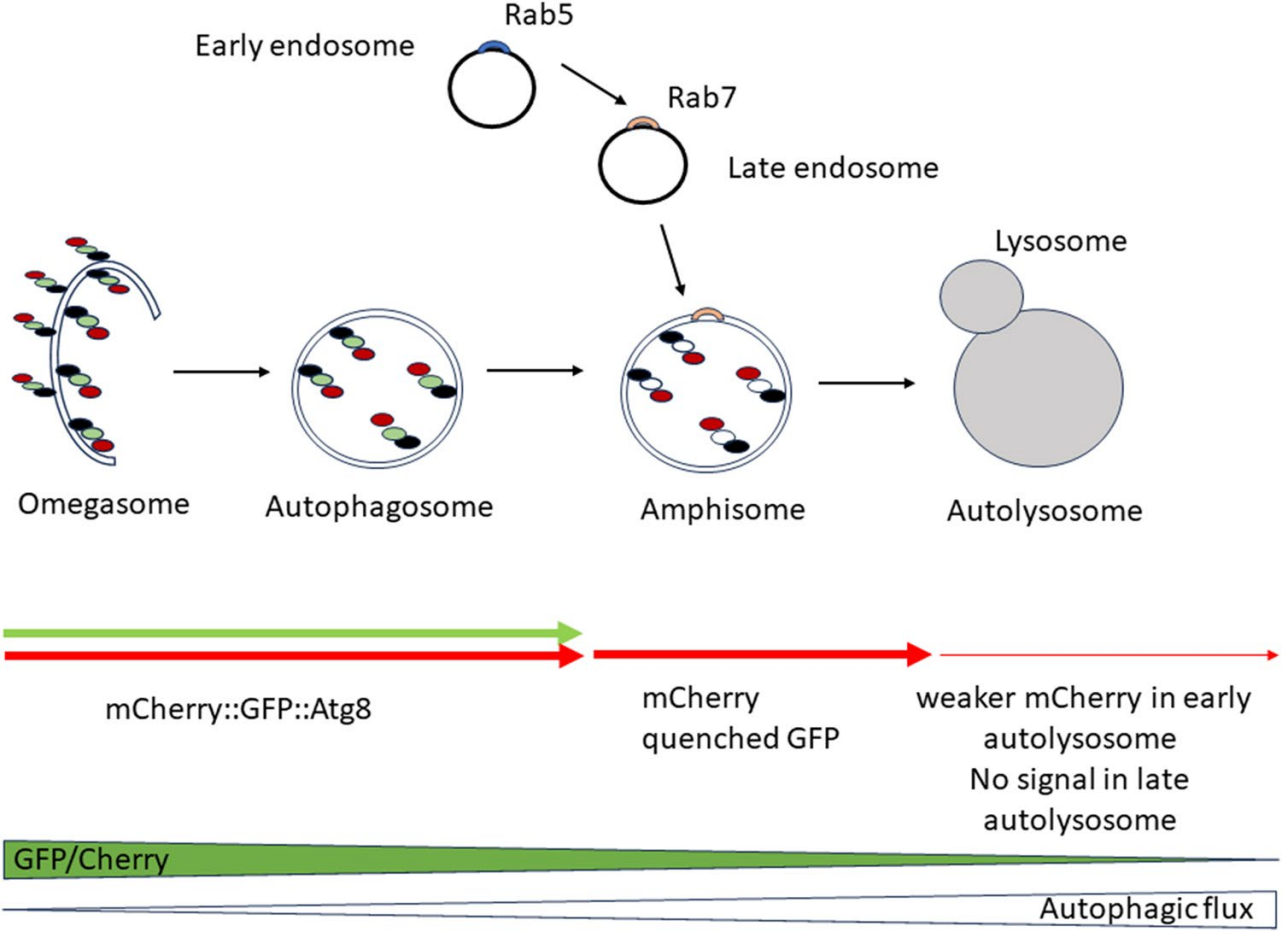
Diagram of autophagy. Atg8 binds to the endoplasmatic reticulum membrane and starts to form a cup-shaped double-membrane structure called omegasome, and then a vesicle called autophagosome. Some of proteins are transported from Golgi apparatus or plasma membrane in endosomes, which contain Rab5 in their early stage, and Rab7 in their late stage. Autophagosome may fuse with the late endosome to form an amphisome. In the last step, after fusion with the lysosome, an autolysosome is formed, and the cargo of the vesicle is degraded. Amphisome can skip the degradation pathway and release cargo into the extracellular matrix. Transgenic flies used for tracking autophagic flux contain Atg8 fused with GFP and mCherry. GFP is sensitive to pH changing, which means that green fluorescence is visible only in autophagosomes. mCherry is more resistant to lower pH, and the red signal is visible from early stages of autophagosomes to Atg8 protein degradation in autolysosomes. A high GFP/mCherry signal ratio indicates low autophagy flux, while a low ratio indicates high autophagy flux.

Autophagy is an important cellular response to starvation and stress. It plays an important role in development, cell death, aging, immune responses and cancer. In general, it is a mechanism used for the degradation of cell organelles and long-lived proteins. One of the best-described autophagy pathways is macroautophagy, here referred to as autophagy. It depends on several proteins encoded by *Autophagy-related genes* (*Atg*s), which are involved in the formation of autophagosomes^1–5^. In the first step, the kinase Atg1 forms complexes with Unc76 and Atg13 and phosphorylates them^6^. Then, these complexes bind to the endoplasmatic reticulum membrane and start to form an omegasome, a cup-shaped double-membrane structure developed in Atg8- and Atg9-dependent manner^7,8^. In the next step, cytosolic components are sequestered inside double-membrane vesicles, called autophagosomes. During the maturation step, the autophagosome is fused with the late endosome to form an amphisome. This process requires Rab GTPases located in the endosome, which interact with a tethering complex on the autophagosome membrane. Rab5 is specific for early endosomes, whereas Rab7 for late endosomes^9,10^. Endosome maturation is characterized by increased luminal acidification and Rab conversion, which is the switch from Rab5 to Rab7^11^. Formed amphisomes fuse with lysosomes, where their inner membrane and contents are degraded by lysosomal hydrolases and subsequently released to the cytosol for recycling (Fig. 1)^12,13^. Autophagy induced by the structural remodeling of the cell increases the level of nutrients and energy and removes damaged elements^14^. Interestingly, there is a population of amphisomes that has a non-degradative function, and is a part of an unconventional pathway of autophagy-related secretion, called secretory autophagy. This process requires Atg5, Atg8, and many Rab proteins. It has been described mostly in the epithelial cells which secret mucus granules or interferon, but also in cells producing interleukin 1β^15–17^.

Autophagy in the brain plays an important role during development^18^, however, in adults, it is also necessary for normal brain functioning and maintaining neuronal plasticity^19^. Atg7 is essential for membrane trafficking and turnover in axons^19^, it also regulates terminal volume, and the kinetics of neurotransmitter release^20^. *Atg* genes are expressed at different levels during the day and night in the mouse liver, skeletal muscles, heart and kidney^21^, and in fruit flies in the whole brain^22^ and in sorted-out glial cells^23^, suggesting the circadian regulation of this process. Autophagy flux in the liver correlates with feeding time, it is the highest in the afternoon, decreases during the night, and increases again in the morning^21^. Moreover, autophagy is involved in the regulation of circadian rhythms in energy homeostasis^24,25^, metabolism, organelle remodeling^26^, daily neuronal plasticity and behavior^22,23^. Circadian changes in behavior, metabolism and gene/protein expression are regulated by the central clock as well as by peripheral oscillators located in the cells of many tissues. In *Drosophila,* the most important cells of the central clock are the small ventral lateral neurons (sLNvs)^27–33^, located in the accessory medulla, which terminate in the dorsal protocerebrum. The clock neurons regulate rhythmic changes in behavior, like activity and sleep, in their pattern and level. The sLNvs release from their terminals the neuropeptide Pigment Dispersing Factor (PDF) to communicate with sleep-promoting dopaminergic cells^34^, posterior lateral protocerebrum (PLP) AstA-expressing neurons^35^, and sleep centers located in mushroom bodies. One of the processes involved in the regulation of behavior is the daily structural plasticity of the sLNv terminals. Their arborization complexity changes during the day, with more projections at the beginning of the day than at the beginning of the night^36^. These differences also affect the number of synapses and, in effect, postsynaptic partners during the day^36,37^. This structural plasticity is regulated in a very complex way, through the molecular mechanism of the clock in the pacemaker cells and peripheral oscillators^38,39^. Autophagy is involved in this process since in astrocytes it is necessary to maintain the rhythm, while in epithelial glia to keep its amplitude^23^.

In this study, we used *Drosophila melanogaster* as a model to investigate interactions between the circadian clock and autophagy, as autophagy genes and their regulators (i.e. TOR) are conserved between insects and mammals^40^. These interactions are well described in insects and are relatively easy to study. A broad range of transgenic strains allows the genetic modification of specific cell types and observation of subsequent changes in metabolism or behavior. We focused on the role of *Atg5* and *Atg7* silencing in PDF-expressing cells, because it was previously shown that these two proteins have an impact on rhythms in other cell types, and even though they belong to the same pathway, they can play different roles in the regulation of cell physiology. It is known that autophagy can be rhythmic and drive daily changes in the visual system neurons^22^ and glial cells^23^, but it was not examined in the pacemaker neurons. In the present study we examined the daily oscillations of autophagy in the pacemaker cells and the impact of autophagy disruption on the functioning of the clock cells.

## Materials and methods

### Flies

Flies were maintained under 12 h of light and 12 h of darkness (LD12:12) or constant darkness (DD) conditions at a temperature of 25 °C or for adult-specific experiment at 18 °C during development and at 29 °C during experiments. Flies were maintained on a standard medium containing 60 g of cornmeal (Glutenex), 8 g of yeasts, 50 ml of artificial honey (Vortumnus), 20 ml of molasses (Tofi), 1.5 g of methyl 4-hydroxybenzoate (Sigma-Aldrich) (dissolved in 15 ml of 90% EtOH) per 1 L of water. Colonies were started with the same number of parental insects to avoid overpopulation and develop larvae at standard density. Flies were fed with the same food during rearing and experiments, except of locomotor activity recording. Males, 5–7 days old, were used for experiments.

For locomotor activity and sleep analysis 2 days old males were transferred to glass tubes of the recording system with food composed of 5 g of sugar, 2 g of agar, 0.8 g of yeasts and 0.15 g of methyl 4-hydroxybenzoate (dissolved in 1.5 ml of 90% EtOH) per 100 ml of water.

In this study the following strains of *Drosophila* were used: *Pdf*-Gal4/Cy0 (kindly donated by Dr. Ch. Helfrich-Förster, Wurzburg) crossed with UAS-*Valium10-*GFP (control flies, BDSC no. 35786), for chronic silencing: UAS-*Atg5RNAi* (BDSC no. 34899), UAS-*Atg7RNAi* (BDSC no. 27707)*,* for adult-specific silencing: t*ub*Gal80ts;UAS-*Atg5RNAi, tub*Gal80ts;UAS-*Atg7RNAi* and for confocal imaging: UAS-GFP::mCherry:*:Atg8a* (kindly donated by Dr. A. Szabo, Szeged University), UAS-*Atg8a::*GFP (BDSC no. 51656), UAS-*Rab5*::YFP (BDSC no. 9775), UAS-*Rab7*::YFP (BDSC no. 23641).

### Locomotor activity and sleep

The locomotor activity system (DAMS, Trikinetics) was used to record activity of single flies. This system consists of monitors equipped with infrared light-emitting diodes and detectors, connected to a computer. Each monitor houses 32 small glass-tubes sealed at both ends: one by food and the other by a foam stopper. Every time the fly crosses the infrared beam in the front of emitter/detector pair, a signal is sent to the computer. Males, 2-days old, were transferred to the tubes. Experiments were performed at 25 °C for chronic or at 29 °C for adult-specific silencing of selected genes, 3 days in LD12:12 followed by 5 days in constant darkness. For sleep analysis, data from the second day of recording in LD12:12 were used. The BeFly Excel macro was used to analyze circadian and sleep parameters^41^. Every experiment was repeated 3 times, at least 60 flies in total were used per group.

### Immunohistochemistry

Heads of male flies were collected at specific time points; Zeitgeber Time 2 (ZT2) (2 h after light-on) and ZT14 (2 h after light-off), then fixed in 4% paraformaldehyde in phosphate buffered saline (PBS; pH 7.4) for 1 h and washed in PBS. In the next step, brains were manually isolated. Prepared samples were fixed again for half an hour, washed in PBS and 3 times in 0.2% PBST (PBS with 0.2% TritonX100). After that, brains were incubated in 5% normal goat serum (NGS) for 30 min first at room temperature, and then with mouse primary antibodies against Pigment Dispersing Factor (PDF, 1:500) (Hybridoma Bank) overnight. Afterwards, samples were washed 3 times in 0.2% PBST, and incubated with goat anti-mouse secondary antibodies conjugated with Cy3 (Jackson Immuno Research), diluted 1:500, for 2 h. Finally, brains were washed 3 times in 0.2% PBST, and 10 min in PBS. Then, they were mounted in Vectashield medium (Vector) and examined with a Zeiss 780 Laser Scanning Microscope.

### Sholl analysis

To visualize projections of sLNv axons, whole brain confocal images were used. Pictures were taken using 40 × lens with an optical zoom of two. Stacks between seven and 19 images were projected in the *x*–*y* axis to obtain a reconstruction of the full trajectory of the axons. The Sholl’s method in ImageJ software was used to quantify the axonal arbors in the dorsal protocerebrum. Concentric rings, centered at the point where the first dorsal ramification opens up, were drawn on each brain hemisphere. The number of intersections of each projection with a particular ring was counted. The total number of intersections was compared between two time points, ZT2 and ZT14 (according to Ref.^36^). Every experiment was repeated 3 times, with at least 10 brains per one group.

### Autophagosome analysis

To study autophagy on the cellular level, specifically in the central clock neurons, we expressed Atg8 protein tagged with both mCherry and GFP only in PDF-producing cells. Males, 5–7 days old, of *Pdf* > GFP::mCherry::*Atg8a* strain were collected at the selected time points (ZT1, ZT4, ZT8, ZT13, ZT16, ZT20, where ZT0 means lights-on and ZT12 lights-off). Brains were fixed and isolated, then mounted in Vectashield medium (Vector). Loss of GFP fluorescence in acidic environment allows observing autophagosome-lysosome fusion. Green or red fluorescence was monitored using confocal microscopy. Fluorescence intensity was measured separately in cell bodies and processes of sLNvs and lLNvs and compared between time points. Green fluorescence indicates early autophagosomes, while red is present at every stage of these vesicle formation. Analysis of GFP/mCherry ratio allows to tract autophagy flux changes. Strong green signal indicates high level of early autophagosomes and low flux. GFP is very sensitive to pH changes, and after fusion of autophagosome with endosome or lysosome its signal is quenched. mCherry is more stable, and red signal is visible until degradation in the late autolysosome. Low GFP/mCherry ratio indicates high autophagy flux (Fig. 1). Every experiment was repeated 3 times, with at least 10 measurements per time point. Experiment was performed in LD12:12 and DD.

To visualize autophagosomes outside of sLNv terminals, 5–7 days old males of *Pdf* > *Atg8*::GFP, *Pdf* > *Rab5*::YFP and *Pdf* > *Rab7*::YFP strain were used. Brains were fixed and isolated. Immunostaining with anti-GFP (overnight, 1:1000, rabbit) and secondary goat anti-rabbit Alexa488 conjugated (2 h, 1:1000) was performed.

### Mass spec analysis

Flies, 5–7 days old males, of *Pdf* > *Atg8*::GFP strain were collected at ZT1 and ZT13 (one hour before most extreme changes of the sLNv terminal complexity). Heads were isolated in liquid nitrogen and cryogenically homogenized using pestle to make a powder. Samples of 200 mg of tissue were used for the further analysis (around 1000 individuals per sample). They were dissolved in 500 µl of 250 mM sucrose, 20 mM HEPES, 1 mM EDTA, with freshly added protease inhibitor and centrifuged for 10 min at 580 rpm. Supernatant was transferred to a clean vial. Direct immunoprecipitation was used to isolate autophagosomes produced specifically in LNvs and marked with GFP. First, pre-cleaning of the supernatant was performed by incubation with binding-control magnetic beads (ChromoTek) for 1 h in 4 °C. Proteins which bound to the control beads were removed using magnetic separator (DynaMag). Then ChromoTek GFP-Trap Magnetic (Proteintech) particles were used to pull down GFP-positive vesicles from the sample (according to the manufacturer’s protocol). After washing, the samples were analyzed using mass spec. The analysis was carried out in MS-Lab of the Polish Academy of Sciences. We used wild-type flies as negative controls (non-specific binding), and all proteins which were detected in the negative control samples were removed from the further analysis of experimental data.

### Statistical analysis

Outlier data were detected using Grubbs’ test and removed (significance level = 0.05). Statistical analysis was performed using parametric Dunnett’s multiple comparison test for period of locomotor activity and sleep analysis (experimental group compared with two parental controls), Tukey’s test for autophagosomes measurements or non-parametric Mann–Whitney test to detect differences between two groups in sLNv terminal complexity analysis. GraphPad Prism 6 software was used for analyses. Statistically significant differences were set at p < 0.05. Detailed statistics is provided in the Supplementary Tables S1 and S2.

### Ethics approval

This research used invertebrates as a model. No ethical approval is required.

## Results

### Effect of autophagy in clock neurons on locomotor activity rhythm is adult-specific

PDF-expressing cells belong to the pacemaker neurons responsible for driving circadian rhythms in behavior, affecting period of the locomotor activity rhythm, sleep level and its pattern. Using transgenic flies, we silenced the main autophagy genes in PDF-expressing cells to investigate whether this self-degradative process may affect LNvs physiology. We emphasized the importance of two genes associated with autophagy, *Atg5* and *Atg7*, as it has been previously demonstrated that they exhibit rhythmic expression in both, the whole brain^22^ and clock neurons^42^, and are thereby implicated in the regulation of circadian changes in the morphology of L2 interneurons^22^. First, we used chronic silencing which affected autophagy from embryogenesis through development until adulthood. The obtained results were not conclusive, however, as we observed higher arrhythmicity in flies with *Atg5* silencing (24%), but no effect after silencing of *Atg7* (3%) (Table 1). Flies from both experimental groups were more active during the morning peak of activity, at ZT0 compared with parental strains (Table 1), however, the evening peak of activity and total daily activity were not affected (Table 1).

**Table 1:** Locomotor activity parameters of flies with decreased autophagy in clock neurons LNvs (*Atg5*, *Atg7* silencing).

Driver line	Responder line	Rhythmicity [%]	Period of the rhythm [h]	Morning activity [counts/h], ZT0	Evening activity [counts/h], ZT12	Total activity [min/24 h]	No. of flies
*Pdf*	Valium	99	24.6	90.1	90.4	751.9	86
	*Atg5RNAi*	**76**	24.3	**103.2**	97.1	670.4	96
	*Atg7RNAi*	97	24.3	**103.4**	86.9	742.3	90
*w*^*1118*^	*Atg5RNAi*	93	23.6	65.4	72.0	535.6	60
	*Atg7RNAi*	97	23.4	36.2	71.7	539.6	59
Adult-specific							
* Pdf*	Valium	97	23.3	122.2	141.2	812.8	72
	*tub*Gal80ts; *Atg5RNAi*	**78**	**24.3**	125.6	86.2	672.6	92
	*tub*Gal80ts; *Atg7RNAi*	**68**	**23.7**	112.4	**104.1**	839.1	57
* w*^*1118*^	*tub*Gal80ts; *Atg5RNAi*	98	23.3	122.2	97.6	806.6	81
	*tub*Gal80ts; *Atg7RNAi*	94	22.8	138.2	137.1	827.9	80

The chronic silencing experiment was performed at 25 °C, while the adult-specific at 29 °C. *Pdf* > Valium and heterozygous responder parental lines were used as controls. Increased percent of arrhythmic flies was observed in *Pdf* > *Atg5RNAi, Pdf* > *tubGal80ts;Atg5RNAi* and *Pdf* > *tubGal80ts;Atg7RNAi* strains, while period of the circadian locomotor activity rhythm was affected only in adult-specific *Atg5* and *Atg7* silencing. The morning peak of activity at ZT0 (the number of fly’s passages through the infrared beam per hour) was increased in *Pdf* > *Atg5RNAi* and *Pdf* > *Atg7RNAi* strains, while the evening peak at ZT12 was increased only in *Pdf* > *tubGal80ts;Atg7RNAi* group. Total activity was increased only in *Pdf* > *Atg5RNAi* strain. Detailed statistics is provided in Supplementary Table S1.

Significant values are in bold.

Autophagy is an important process during development of the nervous system, and its disruption at an early stage of life may cause abnormal functions of adult brain. Having this in mind, we used the TARGET system to affect autophagy in an adult-specific manner. Indeed, 3 days of *Atg5* and *Atg7* downregulation in the adult stage decreased rhythmicity by 22% and 32%, respectively. Moreover, the period of the locomotor activity rhythm was significantly longer than in parental lines: for *Pdf* > *tub*Gal80ts;*Atg7RNAi* in 0.4 h, and 1 h for *Pdf* > *tub*Gal80ts;*Atg5RNAi* (Table 1). Total activity and the morning peak of activity in flies with autophagy disruption were not affected (Table 1), however, flies with *Atg7* silencing were more active at ZT12, during the evening peak of activity (Table 1).

### Autophagy in clock neurons maintains siesta time

**Figure 2:**
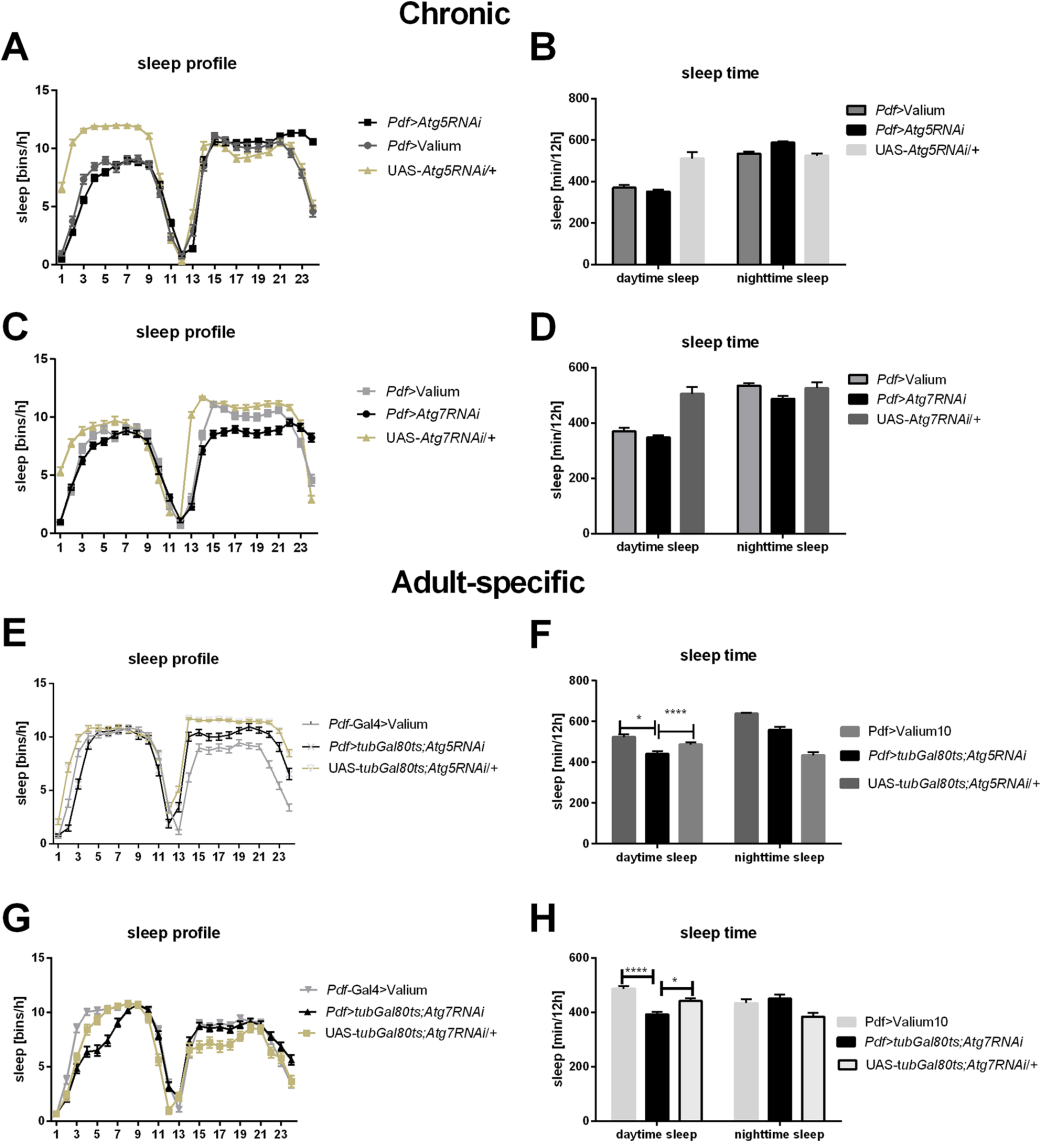
Sleep changes observed after autophagy disruption in the pacemaker cells. (**A**–**D**) chronic silencing of *Atg5* (**A**,**B**) and *Atg7* (**C**,**D**) gene expression in PDF-producing neurons did not affect total sleep time, but changed the pattern of sleep during dusk and in the first part of the day. (**E**–**H**) adult-specific *Atg5* (**E**,**F**) and *Atg7* (**G**,**H**) silencing in clock neurons affected daytime sleep, with no changes in nighttime sleep. Statistically significant differences are marked with asterisks: *p ≤ 0.05, ****p ≤ 0.0001.

Analysis performed in flies with chronic silencing of *Autophagy-related gene 5* in the pacemaker showed increased sleep amount at the end of the night, one hour before lights-on (Fig. 2A), however, total sleep time was not changed (Fig. 2B). Chronic silencing of *Atg7* decreased the number of 5-min bins/hour during the night, between ZT15 and 21, and just before lights-on (Fig. 2C), however, also in this case amount of total sleep was not affected (Fig. 2D).

On the other hand, adult-specific experiments showed that decreased autophagy levels affect daytime siesta, after both *Atg5* and *Atg7* silencing in PDF-expressing cells. After *Atg5* silencing, siesta was delayed and reached 10 bins/h one hour later than in the control, at ZT5 (Fig. 2E). We observed a decreased length of sleep during the day compared with controls (Dunnett’s test: *Pdf* > *tub*Gal80ts*;Atg5RNAi* vs *Pdf* > Valium p < 0.0001, *Pdf* > *tub*Gal80ts*;Atg5RNAi* vs UAS-*tub*Gal80ts*;Atg5RNAi/* + p < 0.0001) (Fig. 2F). In case of *Atg7* silencing, the pattern of sleep was changed during the day, with decreased amount of bins/h in the middle of the day between ZT4 and ZT7 (Fig. 2G). Total sleep time during the day was also decreased in these flies (*Pdf* > *tub*Gal80ts*;Atg7RNAi* vs *Pdf* > Valium p < 0.0001, *Pdf* > *tub*Gal80ts*;Atg7RNAi* vs UAS-*tub*Gal80ts*;Atg7RNAi*/ + p = 0.0074), while during the night sleep time was not affected (Fig. 2H).

### sLNv axonal plasticity depends on Atg5 function during development and Atg7 in adulthood

**Figure 3:**
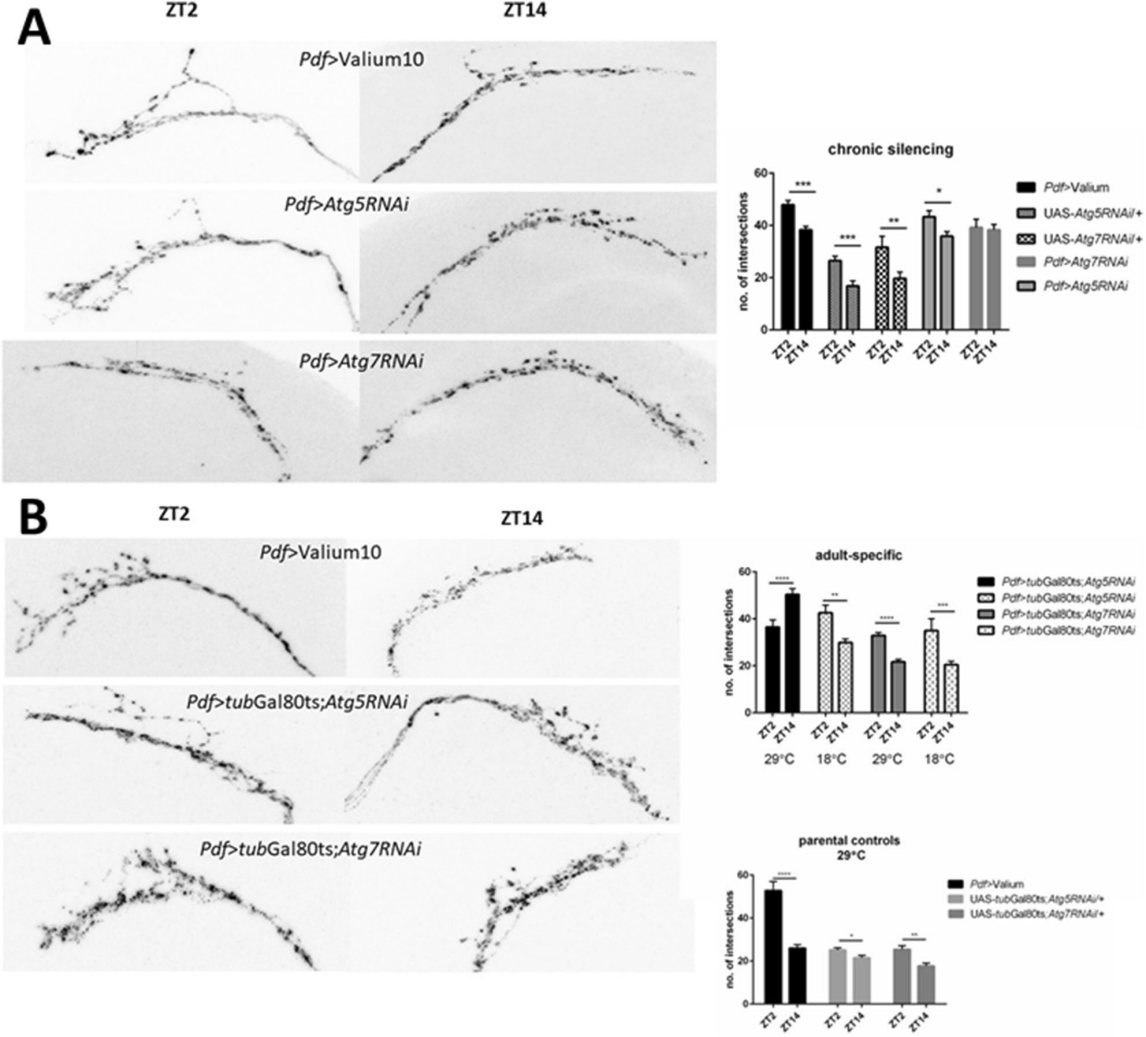
Daily changes in sLNv axonal arborizations. (**A**) chronic autophagy disruption in PDF-producing cells. *Atg5* silencing did not affect the pattern of complexity changes with higher number of intersections at the beginning of the day than during the night, while *Atg7* silencing abolished differences between time points. (**B**) adult specific autophagy disruption in LNvs caused no changes in the daily pattern of arborization after *Atg7* silencing, and reversed pattern after *Atg5* silencing. Experimental flies kept at 18 °C showed normal pattern of arborization, similar to parental strains. Left panel shows representative pictures of processes. Statistically significant differences are marked with asterisks: *p ≤ 0.05, **p ≤ 0.01, ***p ≤ 0.001, ****p ≤ 0.0001.

Activity as well as sleep levels and patterns are regulated by many factors, however, their circadian regulation depends on PDF-expressing neurons. Rhythmic release of the clock neurotransmitter PDF and the daily axonal plasticity of sLNv play the main role in this process. We collected brains at two selected time points (ZT2 and ZT14) and used anti-PDF immunostaining to visualize the pacemaker cell terminals. Sholl analysis showed that at a standard temperature of 25 °C, in control flies the sLNv terminal complexity had normal daily changes, with a higher score at the beginning of the day than during the night (Mann–Whitney’s test: *Pdf* > Valium p < 0.0001, UAS-*Atg5RNAi*/ + p = 0.0005, UAS-*Atg7RNAi*/ + p = 0.0045). Chronic silencing of *Atg5* in LNvs did not affect daily changes in the complexity of sLNv terminals (p = 0.0183), however, *Atg7* silencing disrupted this rhythm with similar number of intersections during the day and night. (p = 0.818) (Fig. 3A).

Adult-specific experiments were performed at 29 °C, in both parental and experimental flies. Control flies showed normal daily changes in the complexity of sLNv terminals with higher complexity of sLNv terminals at the beginning of the day (Mann–Whitney’s test: *Pdf* > Valium p < 0.0001, UAS-*tub*Gal80ts;*Atg5RNAi*/ + p = 0.0256, UAS-*tub*Gal80;*Atg7RNAi*/ + p = 0.0024). Because the number of intersections varied between parental strains, in order to exclude the possible effect of genetic background we performed additional control, which was *Pdf* > *tub*Gal80ts;*Atg5RNAi* and *Pdf* > *tub*Gal80ts;*Atg7RNAi* kept at 18 °C. In this temperature Gal80 is active and blocks Gal4, which prevents RNAi expression. We observed normal daily pattern in the sLNv terminals complexity in these strains (p = 0.023 for *Pdf* > *tub*Gal80ts;*Atg5RNAi* and p = 0.0027 for *Pdf* > *tub*Gal80ts;*Atg7RNAi*). In our experimental strains after 3 days of *Atg7* silencing in the clock neurons, the daily pattern of changes in their terminal arborization was not affected (p < 0.0001), however, *Atg5* silencing reversed the pattern of changes, with more intersections during the night than during the day (p = 0.008) (Fig. 3B).

### Autophagic activity shows daily changes in both cell bodies and processes of clock neurons

**Figure 4:**
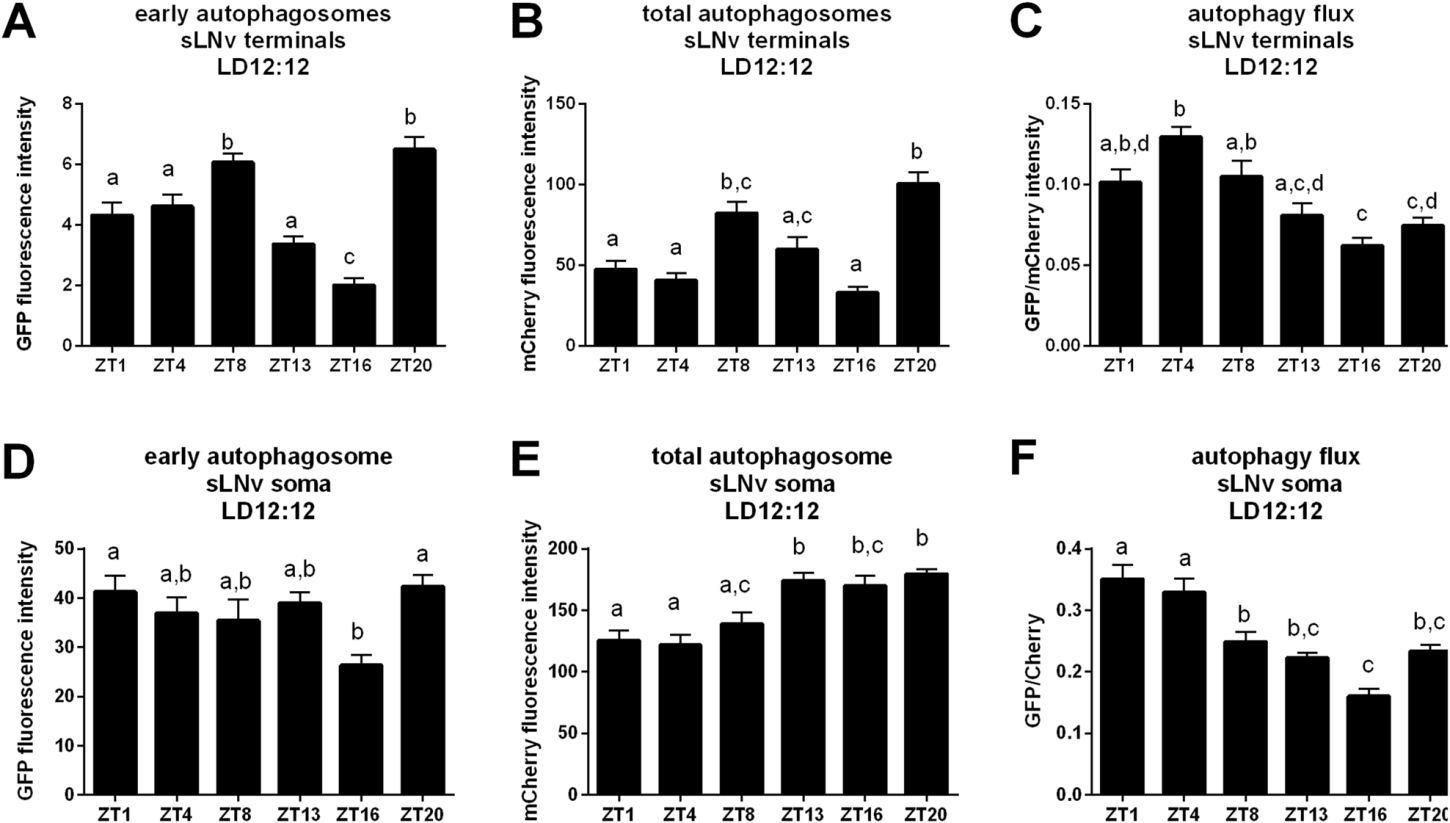
Daily changes of autophagy level in sLNv. The fluorescence intensity was measured in the terminals and in somata of sLNv at selected time points (ZT1-ZT20). Green fluorescence represents the level of early autophagosomes, red fluorescence – total autophagosomes. Autophagy flux was measured as GFP/mCherry ratio. Statistically significant differences are marked with different letters, where the same letter above two or more bars means no statistically significant differences between these groups. Detailed statistics is provided in Supplementary Table S2.

PDF-expressing neurons show daily changes in their physiology, such as rhythmic changes of protein levels, neurotransmitter release, branching of terminals and changes in synaptic partners. Some of these processes might be driven by rhythmic autophagy. To check whether autophagy activity in LNvs shows daily changes, we induced GFP::mCherry::Atg8a expression in LNvs. Using whole brain imaging, we were able to visualize the green fluorescence of early autophagosomes (EA) and the red signal for all autophagosomes and autolysosomes (total, TA). We measured fluorescence intensity separately for sLNv and lLNv in cell bodies and processes. The results showed the daily differences in autophagosome processing in sLNv terminals – EA had a stronger signal in the evening, at ZT8 and at the end of the night, at ZT20, just after the minimum at ZT16 (Fig. 4A). A similar pattern was observed for the fluorescence signal for TA—the highest at the end of the day, at ZT8 and at the end of the night, at ZT20 (Fig. 4B). Autophagy flux, which is defined as autophagic degradation activity, reached the maximum at ZT4 and minimum during the night (Fig. 4C). This rhythm seems to be driven by the clock and light together, as in constant darkness (DD) the level of early autophagosomes reached the maximum at CT8 only (Fig. S1A), higher level of total autophagosomes was observed between CT4 and CT13 and again at CT20 (Fig. S1B), and in effect autophagy flux showed maximum at the beginning of the subjective day, at CT1 and during the subjective night, at CT16 (Fig. S1C).

The daily pattern of early autophagosome abundance in the cell bodies of sLNvs was similar to that observed in the terminals, with the minimum at ZT16 (Fig. 4D). A different pattern was observed for total autophagosome quantity in the sLNv somata, with less TA during the day and more autophagosomes during the night (Fig. 4E). Autophagy flux was higher during the day, at ZT1 and ZT4, and the lowest in the middle of the night, at ZT16 (Fig. 4F).

**Figure 5:**
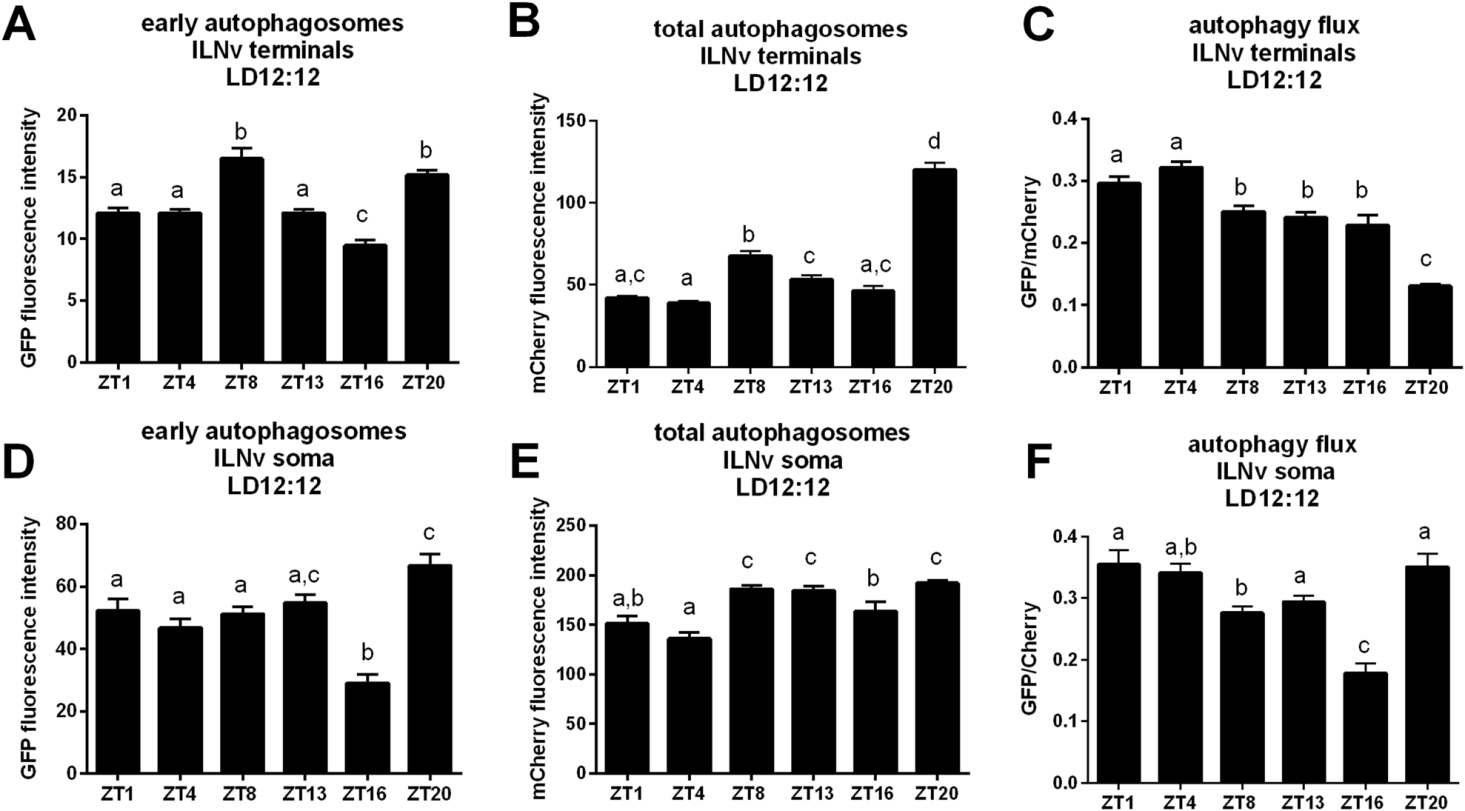
Daily changes of autophagy level in lLNv. Whole brains of *Pdf* > GFP::mCherry::*Atg8a* flies were isolated at selected time points (ZT1-ZT20). The fluorescence intensity was measured in the terminals and somata of lLNv, respectively. Green fluorescence intensity represents the level of early autophagosomes, red fluorescence – early and late (total) level of autophagosomes. Autophagy flux is shown as GFP/mCherry ratio. Statistically significant differences are marked with different letters. Detailed statistics is provided in Supplementary Table S2.

In lLNv processes in the medulla, the abundance of early autophagosomes peaked twice a day, during the day, at ZT8 and at the end of the night, at ZT20 (Fig. 5A). A similar pattern was observed for total autophagosome abundance, with a higher score at ZT8 and a maximum at ZT20 (Fig. 5B). Autophagy flux was higher at the beginning of the day, at ZT1 and ZT4, and the minimum was observed at the end of the night, at ZT20 (Fig. 5C). Interestingly, in constant darkness GFP signal was very low, at the level of background, while RFP signal was 10 times lower than in LD12:12 conditions. Daily pattern of total autophagosomes showed the highest score in the middle of the subjective day, at CT4 and the minimum at the end of the subjective night, at CT20 (Fig. S2A).

The daily pattern of EA in the lLNv cell bodies was similar to that observed for sLNv somata, with the minimum in the middle of the night, at ZT16 (Fig. 5D). Total autophagosome quantity in the lLNv cell bodies was higher at the end of the day and during the night (Fig. 5E). Autophagy flux showed minimum in the middle of the night, at ZT16 (Fig. 5F). In constant darkness early autophagosomes level was the highest at the end of the subjective day, at CT8 and at the end of the subjective night, at CT20 (Fig. S2D), while total autophagosome was similar during the subjective day and subjective night, except of CT1, when it showed a minimal score (Fig. S2D). Autophagy flux in the lLNv somata in DD was the highest in the subjective morning, and the lowest at the beginning of the subjective night (Fig. S2F).

### Atg8-positive vesicles are observed outside of the sLNv terminals

**Figure 6:**
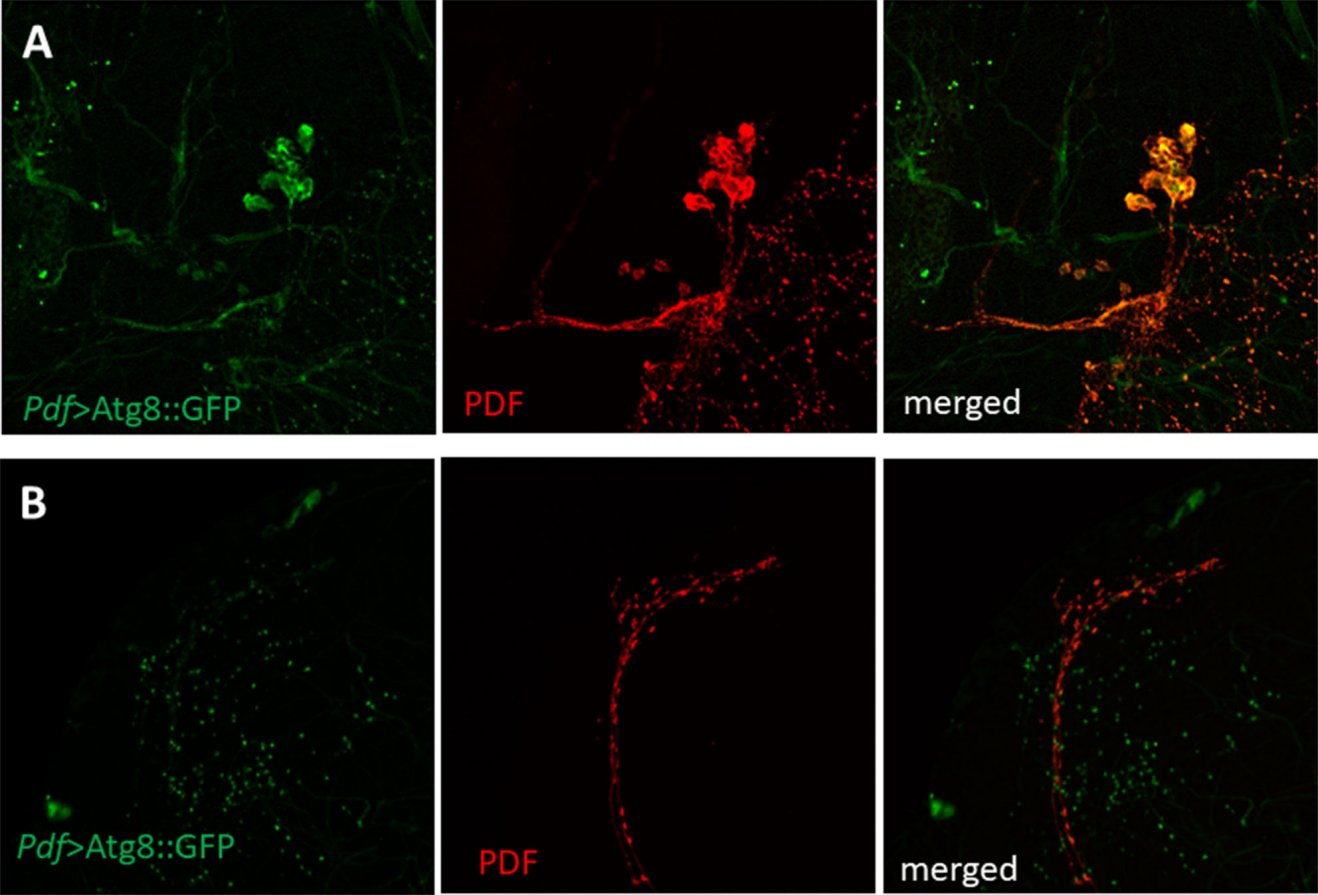
Fluorescence signal of Atg8::GFP protein in LNvs is located in the cell bodies (**A**) where it co-localizes with anti-PDF immunostaining and outside of sLNv terminals (**B**).

During our experimental procedure we used different protocols to obtain the best signal of autophagosomes under the confocal microscope. Surprisingly, using the *Pdf* > *Atg8*::GFP strain and anti-GFP immunostaining, we observed small fluorescent dots in the dorsal brain but outside the sLNv terminals, and we did not detect similar signal in the proximity of cell bodies (Fig. 6). This signal was observed at all time points examined, and it was stronger during the day than during the night (Supplementary Fig. S3).

**Figure 7:**
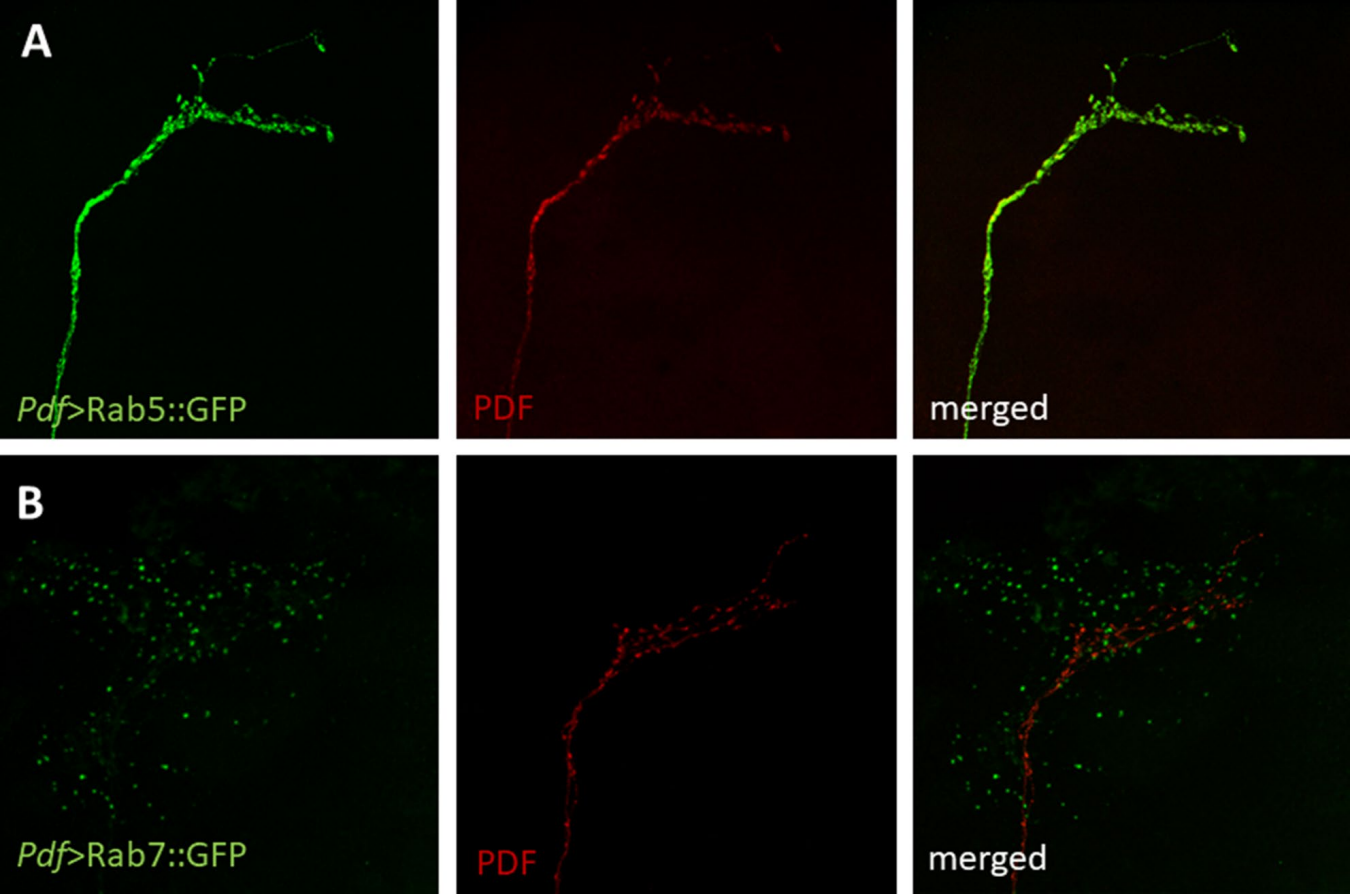
Late endosomes are released from sLNv terminals. Fluorescence signal of early exosomes marked with Rab5::YFP is visible inside sLNv processes immunostained with anti-PDF (**A**), while late exosomes marked with Rab7::YFP are found outside terminals (**B**). Brains were collected at ZT1, immunostained with anti-GFP (green) and anti-PDF (red).

We did not observe this phenomenon in the *Pdf* > GFP::mCherry:*:Atg8a* strain, however, it is possible that this fusion protein is too large to be involved in vesicle release from the cell or that the signal is too weak to be detected without immunostaining. Autophagosomes could be removed from cells by secretory autophagy, however, this process is largely unknown, and has not been described in *Drosophila* in details. To confirm specificity of our staining, we expressed Rab5::YFP and Rab7::YFP in PDF-expressing neurons, which allow to recognize early and late exosomes, respectively. Indeed, we observed Rab7, but not Rab5 signals outside the sLNv terminals (Fig. 7). Because in some strains non-specific fluorescent signal was observed, we used parental strains (*Pdf*-Gal4 and UAS-GFP::*Atg8a*) immunostained with anti-GFP and anti-PDF as additional controls and we did not observe any signal in the green channel (Supplementary Fig. S4).

### Autophagosome cargo in LNvs changes during the day

The results described above show that autophagy in PDF-expressing neurons is rhythmic, however, daily changes could be detected not only in autophagic activity but also in its target. To investigate potential differences in the autophagosomes cargo during the day and night, we isolated Atg8::GFP vesicles produced in LNvs at two time points – ZT1 and ZT13 and identified proteins using mass spectrometry. We obtained a list of 280 proteins that were present in the samples (Table S3). We compared protein composition between time points and divided them into groups detected only at specific time point. Interestingly, only 21 proteins were present at ZT13 but not at ZT1 (Table S3). PANTHER analysis (patherdb.org) depicted also extracellular proteins in the samples: 22 specifically at ZT1, 3 specifically at ZT13 and 5 proteins showed up at both time points (Table 2).

**Table 2:** Extracellular proteins detected as autophagosome cargo: only at ZT1, only at ZT13 and at both time points.

ZT1	ZT13	Both
Vago	Obp83a	Ferritin 2LCH
CREG	CG42325	Growth blocking peptide 2
Chitinase 2	Chemosensory protein A75a	yellow-f2
Neural Lazarillo		Niemann-Pick type 2 h
IP12536p		antennal protein 10
Sod3		
Serpin 43Ab		
Lsp1gamma		
Acetylocholinesterase		
Serpentine		
CG16820		
Spn77Bc		
Tolkin		
Ribonuclease X25		
Angiotensin converting enzyme		
Peritrophin-A		
Amalgam		
Obp83g		
Nimrod B2		
Serine protease homolog 242		
Fondue		
Persephone		

According to PANTHER 18.0 analysis.

## Discussion

Although autophagy is mostly studied in the context of stress and pathology, in the nervous system it is a physiological process that occurs constitutively under normal conditions. In mice hippocampal neurons autophagosomes are continuously generated in the distal axon, and less frequently in dendrites and soma^43^. The significance of this process is highlighted by the fact that its deficiency in neurons induces neurodevelopmental disorders^44^ and neurodegeneration in adult life^45,46^. During neurogenesis it is involved in axon outgrowth and guidance^47^, dendritic growth and branching^48^, synaptic development^49^ and synapse pruning^50^.

The importance of autophagy in the adult brain has been reported for LC3-associated phagocytosis in glia to clear axon debris ^51^. In turn, Atg7 was described as a key protein for neuronal survival^52^ and Atg5 as maintaining the size of the presynaptic sites^53^. Circadian regulation of autophagy gene expression suggests that this process is involved in daily changes in cell physiology^22,23^. It was previously shown that autophagy genes in *Drosophila* are expressed in a clock-dependent manner in the brain, in the sorted-out glial cells, and in the central clock neurons, with different patterns of cycling in each cell type^22,23,42^.

The sLNvs are major clock neurons involved in the regulation of locomotor activity and sleep, as well as other daily physiological and behavioral processes. Four out of five sLNvs produce the clock neurotransmitter PDF, which is transported and released in a circadian manner from their terminals in the dorsal brain^54^. These terminals show strong daily neuronal plasticity, with the highest complexity of the axon structure at the beginning of the day and the simplest form at the beginning of the night^36,37^. This process is regulated by the pacemaker cells and peripheral clocks located in the glia^23,36,38^. The precise mechanism of this remodeling is still unknown, however, an appropriate PDF expression level is necessary to maintain this rhythm^55^. The key players are also matrix metalloproteases Mmp1 and Mmp2, which promote the reduction of arborization complexity^55^. The daily neuronal and synaptic plasticity is very important as it forces changes in synaptic partners throughout the day^37^. Modifications of terminal shape and length require reorganization of the cytoskeleton and cell membranes and may involve autophagy. Using chronic modulation of autophagy gene expression, we showed that this process is necessary for the normal formation of sLNv projections or the appropriate functioning of the regulatory mechanisms. Atg7 seems to be a crucial factor during the brain development, as we did not observe daily changes of axon terminals in flies with chronic *Atg7* silencing, however, an adult-specific decrease of *Atg7* expression for 3 days did not show any changes in the complexity pattern. On the other hand, chronic *Atg5* silencing did not induce any changes in this rhythm, while in the adult-specific experiment, the *Pdf* > *tub*Gal80ts*;Atg5RNAi* flies showed a reversed pattern of daily changes in the sLNv terminals complexity. This result suggests that Atg5 and Atg7 play different roles, with Atg7 having a higher importance during development and Atg5 in adult life, at least in the regulation of clock neuron arborization of axons. The different functions of Atg5 and Atg7 in circadian plasticity were already described in L2 interneurons in the first optic neuropil (lamina). Chronic *Atg5* silencing disrupts daily changes in the size of L2 dendritic trees, while after *Atg7* silencing, the rhythm is still observed, but its pattern is changed^22^. Importantly, autophagy disruption did not cause cell death, as LNvs morphology was normal, cells still produced and released PDF, and flies were rhythmic, which suggests that pacemakers were functional.

Our results suggest that chronic decrease of autophagy in pacemaker cells may increase response to light. Experimental flies were more active after lights-on than controls. In addition, sleep time at the end of the night (ZT22-24) was increased, but total activity was not affected. On the other hand, the adult-specific decrease of autophagy did not affect activity and sleep during the night, but flies showed shorter siesta time in the morning.

Surprisingly, changes in the daily pattern of sLNv terminal complexity after chronic disruption of autophagy did not correlate directly with sleep pattern changes. *Atg5* silencing did not change sLNv arborization pattern of terminals, while after *Atg7* silencing this rhythms was not maintained. Both modifications, however, increased morning peak of activity and did not affect total sleep level. On the other hand, disruption of autophagy in adults, both by *Atg5* and *Atg7* silencing, affected siesta time, but again, the effect on sLNv terminal complexity was different. Changes in the complexity of sLNv terminals during the day seem not to drive sleep directly. Moreover, higher complexity during the night was connected with longer sleep time during the night after silencing of autophagy genes in glia^23^. Indeed, this regulation mechanism is more complex, and autophagy is only one of many players. In the future investigation it would be good to check which synaptic partners are changed throughout the day^37^ and which signaling pathway is disrupted after autophagy silencing. This issue seems to be more important for sleep regulation than changes in axon complexity by itself.

Clock neurons show strong rhythmicity in their physiology. It was not surprising that also autophagy in these cells shows daily changes. This phenomenon was detected by tracking autophagosome abundance throughout the day in the somata and terminals of selected cells. We showed that the daily pattern differs between sLNvs and lLNvs and also between somata and terminals. Detailed analysis of the autophagy pattern in the sLNv terminals showed that intense vesicle formation starts at the end of the night, and at the end of the day, and autophagy flux is the most intense around noon. This pattern suggests that autophagy is correlated with remodeling of the terminals, with strongly arborized terminals in the morning, which start to shrink later during the day, when higher autophagic activity is observed.

In lLNvs cell bodies, the daily pattern in autophagosome abundance was similar to that observed for sLNvs. The quantity of early autophagosomes in the terminals had two peaks at the end of the day and at the end of the night, however, the abundance of total autophagosomes was changing daily, with the highest score at ZT20. That means higher autophagy activity in terminals during the day and the lowest at ZT20, while in the somata the flux is inhibited in the middle of the night. Differences in the autophagy pattern between sLNvs and lLNvs were described on the transcriptional level^42^.

Interestingly, light seems to regulate autophagy in clock neurons, since in constant darkness the pattern of daily changes in early and late autophagosomes was different that in LD12:12 and autophagy flux was higher. However, we observed very low level of early autophagosomes in the lLNv terminals in the medulla of flies kept in constant darkness, which correlates with the data which showed that autophagy is light-driven in the retina^56^ and may be enhanced by light in hippocampal cells *in vitro*^57^. This phenomenon needs more exploration in the future.

It was previously shown that autophagy in healthy neurons plays an important role. Many autophagosomes are formed in the distal part of axons and are retrogradely transported to the soma where they are fused with lysosomes^58^. This transport is required to relocate neurotrophins and their receptors within the cell body and induce neuronal arborization. Indeed, *Atg5* mutant mice showed decreased neuronal complexity^59^. In addition, autophagy is involved in controlling axonal calcium storage in the endoplasmic reticulum (ER) and, in effect, excitatory neurotransmission^60^. Autophagy is also important for synapses, both at the pre- and postsynaptic sites. Chronic deficiency of autophagy affects dopaminergic neuron terminals, increasing their size, dopamine release and rapid presynaptic recovery, while TOR inhibition increases the number of autophagosomes in axons, decreases the number of synaptic vesicles and attenuates the release of dopamine^20,61^. There is also an evidence that Atg5 can bind to Basoon, a key protein for fusion of synaptic vesicles with the presynaptic membrane and the release of neurotransmitters. In effect, autophagy actively degrades synaptic vesicles or their components^62^. Our results showed that autophagy during development is necessary to drive the plasticity of sLNv terminals, however, this does not apply for Atg5. Moreover, autophagy in the adult brain seems to be more important for daily neuronal plasticity with the use of Atg5.

In addition to protein degradation, autophagy may play a role in the secretion of proteins, like interleukin and galectins, or in inserting proteins into the plasma membrane^63,64^. This process has not been precisely described, yet. It needs Atg5 and interactions of autophagosomes with Sec22b, a SNARE protein anchored on the outer membrane of secretory autophagosomes with syntaxin 3/4 and SNAP-23/29 on the plasma membrane^65^. Although many proteins have been shown to be released through secretory autophagy, this process has not been described in *Drosophila,* yet. Our observations on the basis of confocal images suggest that this mechanism occurs in clock neurons to secrete unknown cargo outside terminals in the dorsal brain. The observed vesicles contained Atg8 and Rab7, but not Rab5, indicating their late endosome/autophagosome origin. The signal was only observed next to the sLNv terminals in the dorsal brain, but neither in the area of axons and cell bodies, nor in the medulla, where lLNv terminals are located, which strengthens the conclusion that these fluorescent dots specifically mark vesicles released in this area.

The ideal would be to find cargo secreted in the observed vesicles. Analyses of confocal images did not show co-localization between vesicles and PDF, a main neurotransmitter released from sLNv terminals, which suggests that this peptide is not the target for secretory autophagy. Isolation of vesicles marked with Atg8::GFP provided data from all stages of autophagosome formation, however, the obtained list of proteins should also include secreted proteins. We compared autophagosomes cargo at two different time points – in the morning (ZT1) and at the beginning of the night (ZT13), because during this time the robust changes in the complexity of terminals were observed. We focused on these proteins, which showed up only at one time point to find potential factors connected with neuronal plasticity and extremal changes between the day and night. Interestingly, only 21 proteins were detected specifically at ZT13 but not at ZT1, and among them were: Strawberry Notch (Sno), Rab10, 14–3-3ɛ, anon1A3, Actin-42A and calpain B, the proteins known to be involved in neuronal remodeling^66,67^. Sno plays an important role during eye development^68^, however in mice *Sno* knockout shows cortical abnormalities, caused by hypogrowth of the projection fibers and dendrogenesis deficiency^69^. Proteins from 14-3-3 family orchestrate different cellular processes, such as intracellular signaling^70^, exocytosis^71^, neurite outgrowth, axonal guidance^72^, dendritic spine remodeling^73^, synaptic plasticity^74^ and neurotransmitter release^75^. 14-3-3 is also known as a regulator of autophagy, which may bind different Atg proteins and promote or inhibit this process^76^. Calpain B is a calcium-dependent protease involved in neuronal remodeling during development and adulthood^77^. Among autophagy cargo at ZT13 there is also Dynein light chain roadblock – a key protein in transport of lysosomes and retrograde signaling vesicles^78^.

More proteins appeared at the cargo list at ZT1: tropomyosin, mitochondrial proteins (Trxr2, Letm1, ND-51N1, Roe1, mRpL22), ubiquitin pathway factors (Ubqn, Uba5, hiw, Prosbeta4), proteins involved in the formation of RNA exosomes (Rrp40), late endosomes (Lamtor5, Vta1), etc., suggesting that during the day more processes required autophagy. Moreover, proteins involved in axon growth were also found in autophagosome cargo at ZT1. One of the examples is Highwire (hiw)—an E3 ubiquitin ligase which restrains neuromuscular junction growth. Selective autophagy regulates hiw protein abundance, which in turn, promotes the synaptic development^49,79^. Cyclical autophagic hiw degradation in PDF-expressing neurons could enhance axon terminal plasticity.

We have also detected proteins that are recognized as extracellular, like Ance (angiotensin converting enzyme), neural Lazarillo (involved in the regulation of behavior)^80^, as well as membrane proteins, like GlyT (glycine transporter) and tetraspanin 96f. Autophagosome cargo composed of extracellular or membrane proteins indicate that they are released through secretory autophagy. In addition, we identified a protein encoded by CG14275, that is predicted to be involved in the regulation of voltage-gated potassium channels and in sleep regulation (according to FlyBase). According to DAVID (Database for Annotation, Visualization and Integrated Discovery) at least 16 scores on the autophagosome cargo list at ZT1 and 7 at ZT13 belong to the group of secreted proteins.

Our results indicate that autophagy in clock neurons has a daily rhythm, that is specific for cell type and location. Changes in autophagosome formation and processing in the sLNv terminals are related to structural remodeling, and possibly late autophagosome content is released outside the cell through secretory autophagy. The mechanism of this process needs further detailed examination, however, we described a new, fascinating mechanism involved in the regulation of circadian neuronal plasticity in clock cells.

### Supplementary Information

Supplementary Figures.

Supplementary Table S1.

Supplementary Table S2.

Supplementary Table S3.

## Data Availability

The datasets generated during the current study are available in Rodbuk UJ at the: 10.57903/UJ/KGYAJH.
