# Supplementary material for: Autophagy as a new player in the regulation of clock neurons physiology of Drosophila melanogaster
Source: Sci Rep. 2024 Mar 13;14:6085. doi: 10.1038/s41598-024-56649-3 (PMC10937918; doi:10.1038/s41598-024-56649-3)
Supplement: Supplementary file 1 — Supplementary Figures. [file 41598_2024_56649_MOESM1_ESM.docx]

**Supplementary Figures**


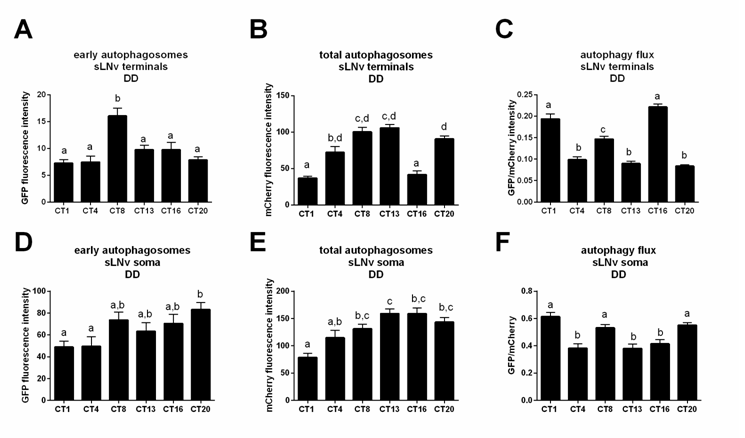


Figure S1 Daily changes of autophagy level in sLNv in constant dark conditions. The fluorescence intensity was measured in the terminals and in somata of sLNv at selected time points (CT1-C20). Green fluorescence represents the level of early autophagosomes, red fluorescence – total autophagosomes. Autophagy flux was measured as GFP/mCherry ratio. Statistically significant differences are marked with different letters, where the same letter above two or more bars means no statistically significant differences between these groups. Detailed statistics is provided in Supplementary Table S2.





Figure S2 Daily changes of autophagy level in lLNv in constant dark conditions. The fluorescence intensity was measured in the terminals and in somata of lLNv at selected time points (CT1-C20). Green fluorescence represents the level of early autophagosomes, red fluorescence – total autophagosomes. Autophagy flux was measured as GFP/mCherry ratio. Statistically significant differences are marked with different letters, where the same letter above two or more bars means no statistically significant differences between these groups. Detailed statistics is provided in Supplementary Table S2.


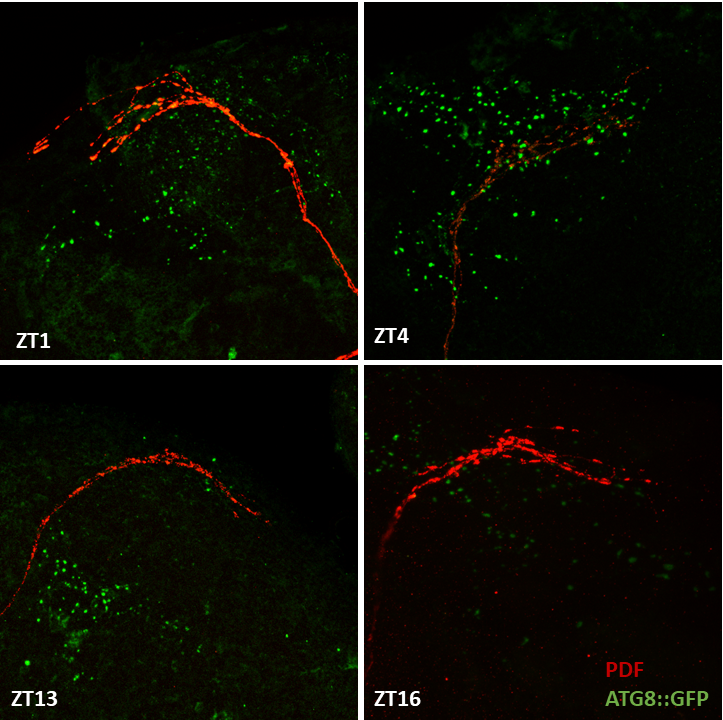


Figure S3 Fluorescence signal of Atg8::GFP protein detected outside of sLNvs terminals at different time points. Immunostaining anti-GFP (green) and anti-PDF (red).


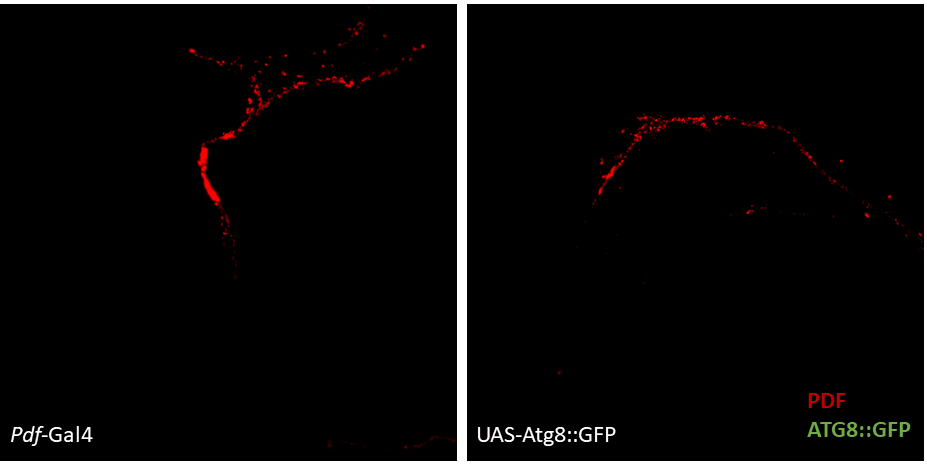


Figure S4 Parental strains (Pdf-Gal4 and UAS-GFP::Atg8) do not show non-specific green signal. Immunostaining anti-GFP (green) and anti-PDF (red).
