# Supplementary material for: Autophagy as a new player in the regulation of clock neurons physiology of Drosophila melanogaster
Source: Sci Rep. 2024 Mar 13;14:6085. doi: 10.1038/s41598-024-56649-3 (PMC10937918; doi:10.1038/s41598-024-56649-3)
Supplement: Supplementary file 2 — Supplementary Table S1. [file 41598_2024_56649_MOESM2_ESM.docx]

Supplementary Table S1

Detailed statistics for Table 1. In the table there are listed p-value. Experimental genotypes with calculated statistically significant differences (p≤0.05) with both control strains are marked with bold.

| Period of locomotor activity | *Pdf*>Valium | UAS-*Atg5RNAi*/+ | UAS-*Atg7RNAi*/+ | *Pdf*>Valium 29°C | UAS*-tub*Gal80ts;*Atg5RNAi*/+ | UAS-*tub*Gal80ts;*Atg7RNAi*/+ |
| --- | --- | --- | --- | --- | --- | --- |
| *Pdf>Atg5RNAi* | 0.5134 | <0.0001 |  |  |  |  |
| *Pdf>Atg7RNAi* | 0.6931 |  | <0.0001 |  |  |  |
| ***Pdf*>*tub*Gal80ts;*Atg5RNAi*** |  |  |  | <0.0001 | <0.0001 |  |
| ***Pdf*>*tub*Gal80ts;*Atg7RNAi*** |  |  |  | <0.0001 |  | <0.0001 |

| Total activity | *Pdf*>Valium | UAS-*Atg5RNAi*/+ | UAS-*Atg7RNAi*/+ | *Pdf*>Valium 29°C | UAS*-tub*Gal80ts;*Atg5RNAi*/+ | UAS-*tub*Gal80ts;*Atg7RNAi*/+ |
| --- | --- | --- | --- | --- | --- | --- |
| *Pdf>Atg5RNAi* | 0.9831 | 0.1994 |  |  |  |  |
| *Pdf>Atg7RNAi* | 0.8369 |  | 0.987 |  |  |  |
| ***Pdf*>*tub*Gal80ts;*Atg5RNAi*** |  |  |  | 0.0011 | 0.0037 |  |
| *Pdf*>*tub*Gal80ts;*Atg7RNAi* |  |  |  | 0.3588 |  | 0.0045 |

| Morning peak of activity | *Pdf*>Valium | UAS-*Atg5RNAi*/+ | UAS-*Atg7RNAi*/+ | *Pdf*>Valium 29°C | UAS*-tub*Gal80ts;*Atg5RNAi*/+ | UAS-*tub*Gal80ts;*Atg7RNAi*/+ |
| --- | --- | --- | --- | --- | --- | --- |
| ***Pdf>Atg5RNAi*** | 0.0161 | <0.0001 |  |  |  |  |
| ***Pdf>Atg7RNAi*** | 0.0477 |  | <0.0001 |  |  |  |
| *Pdf*>*tub*Gal80ts;*Atg5RNAi* |  |  |  | 0.8872 | 0.895 |  |
| *Pdf*>*tub*Gal80ts;*Atg7RNAi* |  |  |  | 0.2555 |  | 0.0011 |

| Evening peak of activity | *Pdf*>Valium | UAS-*Atg5RNAi*/+ | UAS-*Atg7RNAi*/+ | *Pdf*>Valium 29°C | UAS*-tub*Gal80ts;*Atg5RNAi*/+ | UAS-*tub*Gal80ts;*Atg7RNAi*/+ |
| --- | --- | --- | --- | --- | --- | --- |
| *Pdf>Atg5RNAi* | 0.5175 | 0.0005 |  |  |  |  |
| *Pdf>Atg7RNAi* | 0.8493 |  | 0.1403 |  |  |  |
| *Pdf*>*tub*Gal80ts;*Atg5RNAi* |  |  |  | <0.0001 | 0.4791 |  |
| ***Pdf*>*tub*Gal80ts;*Atg7RNAi*** |  |  |  | <0.0001 |  | 0.0002 |
