# Supplementary material for: Autophagy as a new player in the regulation of clock neurons physiology of Drosophila melanogaster
Source: Sci Rep. 2024 Mar 13;14:6085. doi: 10.1038/s41598-024-56649-3 (PMC10937918; doi:10.1038/s41598-024-56649-3)
Supplement: Supplementary file 3 — Supplementary Table S2. [file 41598_2024_56649_MOESM3_ESM.docx]

Table S2. Detailed statistics for Fig. 4 and 5, S1 and S2. Differences between every time point calculated using Tukey’s test. In the table there are listed p-value, statistically significant differences between two time points are marked with bold.

| **Fig. 4A** | ZT4 | ZT8 | ZT13 | ZT16 | ZT20 |
| --- | --- | --- | --- | --- | --- |
| ZT1 | 0.9924 | **0.0088** | 0.4233 | **0.0020** | **0.0003** |
| ZT4 |  | **0.0278** | 0.1057 | **0.0001** | **0.0012** |
| ZT8 |  |  | **<0.0001** | **<0.0001** | 0.9488 |
| ZT13 |  |  |  | 0.1990 | **<0.0001** |
| ZT16 |  |  |  |  | **<0.0001** |

| **Fig. 4B** | ZT4 | ZT8 | ZT13 | ZT16 | ZT20 |
| --- | --- | --- | --- | --- | --- |
| ZT1 | 0.9695 | **0.0015** | 0.7328 | 0.7423 | **<0.0001** |
| ZT4 |  | **<0.0001** | 0.2042 | 0.9727 | **<0.0001** |
| ZT8 |  |  | 0.1062 | **<0.0001** | 0.2885 |
| ZT13 |  |  |  | 0.1033 | **<0.0001** |
| ZT16 |  |  |  |  | **<0.0001** |

| **Fig. 4C** | ZT4 | ZT8 | ZT13 | ZT16 | ZT20 |
| --- | --- | --- | --- | --- | --- |
| ZT1 | 0.064 | 0.9994 | 0.3987 | **0.0233** | 0.1166 |
| ZT4 |  | 0.1281 | **<0.0001** | **<0.0001** | **<0.0001** |
| ZT8 |  |  | 0.1916 | **0.0730** | **0.0379** |
| ZT13 |  |  |  | 0.6440 | 0.9894 |
| ZT16 |  |  |  |  | 0.9110 |

| **Fig. 4D** | ZT4 | ZT8 | ZT13 | ZT16 | ZT20 |
| --- | --- | --- | --- | --- | --- |
| ZT1 | 0.8910 | 0.7541 | 0.9946 | 0.0204 | 0.9999 |
| ZT4 |  | 0.9992 | 0.9958 | 0.189 | 0.7633 |
| ZT8 |  |  | 0.9637 | 0.4037 | 0.5969 |
| ZT13 |  |  |  | 0.0832 | 0.9698 |
| ZT16 |  |  |  |  | **0.0095** |

| **Fig. 4E** | ZT4 | ZT8 | ZT13 | ZT16 | ZT20 |
| --- | --- | --- | --- | --- | --- |
| ZT1 | 0.9999 | 0.8185 | **<0.0001** | **0.002** | **<0.0001** |
| ZT4 |  | 0.5682 | **<0.0001** | **0.003** | **<0.0001** |
| ZT8 |  |  | **0.0145** | 0.0837 | **0.0021** |
| ZT13 |  |  |  | 0.9995 | 0.9952 |
| ZT16 |  |  |  |  | 0.9651 |

| **Fig. 4F** | ZT4 | ZT8 | ZT13 | ZT16 | ZT20 |
| --- | --- | --- | --- | --- | --- |
| ZT1 | 0.9395 | **0.0007** | **<0.0001** | **<0.0001** | **<0.0001** |
| ZT4 |  | **0.0106** | **0.0001** | **<0.0001** | **0.0006** |
| ZT8 |  |  | 0.9015 | **0.0150** | 0.9859 |
| ZT13 |  |  |  | 0.1778 | 0.9986 |
| ZT16 |  |  |  |  | 0.0706 |

| **Fig. 5A** | ZT4 | ZT8 | ZT13 | ZT16 | ZT20 |
| --- | --- | --- | --- | --- | --- |
| ZT1 | >0.9999 | **<0.0001** | >0.9999 | **0.0107** | **0.0001** |
| ZT4 |  | **<0.0001** | >0.9999 | **0.0062** | **<0.0001** |
| ZT8 |  |  | **<0.0001** | **<0.0001** | 0.3485 |
| ZT13 |  |  |  | **0.0091** | **0.0001** |
| ZT16 |  |  |  |  | **<0.0001** |

| **Fig. 5B** | ZT4 | ZT8 | ZT13 | ZT16 | ZT20 |
| --- | --- | --- | --- | --- | --- |
| ZT1 | 0.9767 | **<0.0001** | 0.0589 | 0.9383 | **<0.0001** |
| ZT4 |  | **<0.0001** | **0.0033** | 0.579 | **<0.0001** |
| ZT8 |  |  | **0.0052** | **<0.0001** | **<0.0001** |
| ZT13 |  |  |  | 0.6128 | **<0.0001** |
| ZT16 |  |  |  |  | **<0.0001** |

| **Fig. 5C** | ZT4 | ZT8 | ZT13 | ZT16 | ZT20 |
| --- | --- | --- | --- | --- | --- |
| ZT1 | 0.3360 | **0.0084** | **0.0007** | **0.0001** | **<0.0001** |
| ZT4 |  | **<0.0001** | **<0.0001** | **<0.0001** | **<0.0001** |
| ZT8 |  |  | 0.8909 | 0.6740 | **<0.0001** |
| ZT13 |  |  |  | 0.9579 | **<0.0001** |
| ZT16 |  |  |  |  | **<0.0001** |

| **Fig. 5D** | ZT4 | ZT8 | ZT13 | ZT16 | ZT20 |
| --- | --- | --- | --- | --- | --- |
| ZT1 | 0.7791 | 0.9999 | 0.993 | **<0.0001** | **0.0113** |
| ZT4 |  | 0.9027 | 0.4137 | **0.0031** | **<0.0001** |
| ZT8 |  |  | 0.9668 | **0.0001** | **0.0057** |
| ZT13 |  |  |  | **<0.0001** | 0.0656 |
| ZT16 |  |  |  |  | **<0.0001** |

| **Fig. 5E** | ZT4 | ZT8 | ZT13 | ZT16 | ZT20 |
| --- | --- | --- | --- | --- | --- |
| ZT1 | 0.3458 | **0.0004** | **0.0007** | 0.7414 | **<0.0001** |
| ZT4 |  | **<0.0001** | **<0.0001** | **0.021** | **<0.0001** |
| ZT8 |  |  | >0.9999 | 0.1398 | 0.9727 |
| ZT13 |  |  |  | 0.1959 | 0.9217 |
| ZT16 |  |  |  |  | **0.0191** |

| **Fig. 5F** | ZT4 | ZT8 | ZT13 | ZT16 | ZT20 |
| --- | --- | --- | --- | --- | --- |
| ZT1 | 0.9930 | **0.0162** | 0.1130 | **<0.0001** | >0.9999 |
| ZT4 |  | 0.0648 | 0.3169 | <0.0001 | 0.9985 |
| ZT8 |  |  | 0.9768 | **0.0048** | **0.0234** |
| ZT13 |  |  |  | **0.0003** | 0.1523 |
| ZT16 |  |  |  |  | <0.0001 |

| **Fig. S1A** | ZT4 | ZT8 | ZT13 | ZT16 | ZT20 |
| --- | --- | --- | --- | --- | --- |
| ZT1 | >0.9999 | **<0.0001** | 0.7119 | 0.7792 | 0.9993 |
| ZT4 |  | **<0.0001** | 0.7845 | 0.8384 | >0.9999 |
| ZT8 |  |  | **0.0004** | **0.0027** | **<0.0001** |
| ZT13 |  |  |  | >0.9999 | 0.8201 |
| ZT16 |  |  |  |  | 0.8780 |

| **Fig. S1B** | ZT4 | ZT8 | ZT13 | ZT16 | ZT20 |
| --- | --- | --- | --- | --- | --- |
| ZT1 | **0.0036** | **<0.0001** | **<0.0001** | 0.995 | **<0.0001** |
| ZT4 |  | **0.0091** | **0.0016** | **0.0155** | 0.2653 |
| ZT8 |  |  | 0.9682 | **<0.0001** | 0.7337 |
| ZT13 |  |  |  | **<0.0001** | 0.3178 |
| ZT16 |  |  |  |  | **<0.0001** |

| **Fig. S1C** | ZT4 | ZT8 | ZT13 | ZT16 | ZT20 |
| --- | --- | --- | --- | --- | --- |
| ZT1 | **<0.0001** | **<0.0001** | **<0.0001** | 0.1052 | **<0.0001** |
| ZT4 |  | **<0.0001** | 0.9434 | **<0.0001** | 0.6679 |
| ZT8 |  |  | **<0.0001** | **<0.0001** | **<0.0001** |
| ZT13 |  |  |  | **<0.0001** | 0.9848 |
| ZT16 |  |  |  |  | **<0.0001** |

| **Fig. S1D** | ZT4 | ZT8 | ZT13 | ZT16 | ZT20 |
| --- | --- | --- | --- | --- | --- |
| ZT1 | >0.9999 | 0.1795 | 0.7872 | 0.4382 | **0.0198** |
| ZT4 |  | 0.2715 | 0.8528 | 0.5375 | **0.0419** |
| ZT8 |  |  | 0.9027 | 0.9996 | 0.8991 |
| ZT13 |  |  |  | 0.9883 | 0.3553 |
| ZT16 |  |  |  |  | 0.8196 |

| **Fig. S1E** | ZT4 | ZT8 | ZT13 | ZT16 | ZT20 |
| --- | --- | --- | --- | --- | --- |
| ZT1 | 0.2170 | **0.0019** | **<0.0001** | **<0.0001** | **<0.0001** |
| ZT4 |  | 0.8666 | **0.0480** | 0.0609 | 0.3688 |
| ZT8 |  |  | 0.2288 | 0.2762 | 0.8976 |
| ZT13 |  |  |  | >0.9999 | 0.8311 |
| ZT16 |  |  |  |  | 0.8555 |

| **Fig. S1F** | ZT4 | ZT8 | ZT13 | ZT16 | ZT20 |
| --- | --- | --- | --- | --- | --- |
| ZT1 | **<0.0001** | **0.2982** | **<0.0001** | **0.0001** | 0.6295 |
| ZT4 |  | **0.0062** | >0.9999 | 0.9816 | **0.0017** |
| ZT8 |  |  | **0.0007** | **0.0290** | 0.993 |
| ZT13 |  |  |  | 0.9623 | **0.0002** |
| ZT1 |  |  |  |  | **0.0081** |

| **Fig. S2A** | ZT4 | ZT8 | ZT13 | ZT16 | ZT20 |
| --- | --- | --- | --- | --- | --- |
| ZT1 | **0.0041** | >0.9999 | >0.9999 | 0.9992 | **<0.0001** |
| ZT4 |  | **0.0028** | **0.0029** | **0.0027** | **<0.0001** |
| ZT8 |  |  | 0.9997 | 0.9919 | **<0.0001** |
| ZT13 |  |  |  | 0.9997 | **<0.0001** |
| ZT16 |  |  |  |  | **0.0009** |
| **Fig. S2B** | ZT4 | ZT8 | ZT13 | ZT16 | ZT20 |
| ZT1 | 0.4008 | **0.0009** | 0.9949 | 0.4268 | **0.0060** |
| ZT4 |  | 0.5317 | 0.6696 | >0.9999 | 0.8020 |
| ZT8 |  |  | **0.0033** | 0.2761 | 0.9959 |
| ZT13 |  |  |  | 0.7171 | **0.0197** |
| ZT16 |  |  |  |  | 0.5751 |

| **Fig. S2C** | ZT4 | ZT8 | ZT13 | ZT16 | ZT20 |
| --- | --- | --- | --- | --- | --- |
| ZT1 | **0.0001** | **<0.0001** | **<0.0001** | **<0.0001** | **<0.0001** |
| ZT4 |  | >0.9999 | 0.9781 | 0.8449 | 0.9960 |
| ZT8 |  |  | 0.9785 | 0.8025 | 0.9671 |
| ZT13 |  |  |  | 0.9945 | 0.6757 |
| ZT16 |  |  |  |  | 0.3750 |

| **Fig. S2D** | ZT4 | ZT8 | ZT13 | ZT16 | ZT20 |
| --- | --- | --- | --- | --- | --- |
| ZT1 | **<0.0001** | **<0.0001** | **<0.0001** | **<0.0001** | **0.0005** |
| ZT4 |  | **0.0002** | **0.0359** | 0.6867 | **<0.0001** |
| ZT8 |  |  | **<0.0001** | **<0.0001** | 0.9259 |
| ZT13 |  |  |  | 0.5656 | **<0.0001** |
| ZT16 |  |  |  |  | **<0.0001** |
